# TGF-β Signaling Pathways in Different Compartments of the Lower Airways of Patients With Stable COPD

**DOI:** 10.1016/j.chest.2017.12.017

**Published:** 2018-04

**Authors:** Antonino Di Stefano, Claudia Sangiorgi, Isabella Gnemmi, Paolo Casolari, Paola Brun, Fabio L.M. Ricciardolo, Marco Contoli, Alberto Papi, Pio Maniscalco, Paolo Ruggeri, Giuseppe Girbino, Francesco Cappello, Stelios Pavlides, Yike Guo, Kian Fan Chung, Peter J. Barnes, Ian M. Adcock, Bruno Balbi, Gaetano Caramori

**Affiliations:** a Divisione di Pneumologia e Laboratorio di Citoimmunopatologia dell’Apparato Cardio Respiratorio, Istituti Clinici Scientifici Maugeri, SpA, Società Benefit, IRCCS, Veruno (NO), Italy; b Centro Interdipartimentale per lo Studio delle Malattie Infiammatorie delle Vie Aeree e Patologie Fumo-Correlate (CEMICEF), Sezione di Medicina Interna e Cardiorespiratoria, Università di Ferrara, Ferrara, Italy; c Modulo di Chirurgia Toracica, Azienda Ospedaliera Universitaria S. Anna, Ferrara, Italy; d Dipartimento di Medicina Molecolare, Università di Padova, Padova, Italy; e Dipartimento di Scienze Cliniche e Biologiche, AOU, Ospedale San Luigi, Orbassano, Università di Torino, Torino, Italy; f Unità Operativa Complessa di Pneumologia, Dipartimento di Scienze Biomediche, Odontoiatriche e delle Immagini Morfologiche e Funzionali (BIOMORF), Università di Messina, Messina, Italy; g Dipartimento di Biomedicina Sperimentale e Neuroscienze Cliniche, Sezione di Anatomia Umana, Università di Palermo, and Euro-Mediterranean Institute of Science and Technology (IEMEST), Palermo, Italy; h Department of Computing and Data Science Institute, Imperial College London, England; i Airways Disease Section, National Heart and Lung Institute, Imperial College London, England; j Priority Research Centre for Lung Health, Hunter Medical Research Institute, New Lambton Heights, NSW, Australia

**Keywords:** airway inflammation, autoimmunity, BAMBI, CTGF, SMAD, TGF-β, BAMBI, bone morphogenetic proteins and activin membrane-bound inhibitor, BMP, bone morphogenetic protein, CCN2, connective tissue growth factor, ECM, extracellular matrix, LAP, latency-associated peptide, LLC, large latent complex, LTBP, latent transforming growth factor-β binding protein, MAPK, mitogen-activated protein kinase, PI3K, phosphoinositide 3-kinase, SMAD, small mother against decapentaplegic, TGF, transforming growth factor, TGFBI, transforming growth factor-β-induced protein, TGF-βR, TGF-β receptor, TGIF, 5′-TG-3′-interacting factor, TRAP-1, transforming growth factor-β receptor-associated binding protein

## Abstract

**Background:**

The expression and localization of transforming growth factor-β (TGF-β) pathway proteins in different compartments of the lower airways of patients with stable COPD is unclear. We aimed to determine TGF-β pathway protein expression in patients with stable COPD.

**Methods:**

The expression and localization of TGF-β pathway components was measured in the bronchial mucosa and peripheral lungs of patients with stable COPD (n = 44), control smokers with normal lung function (n = 24), and control nonsmoking subjects (n = 11) using immunohistochemical analysis.

**Results:**

TGF-β1, TGF-β3, and connective tissue growth factor expression were significantly decreased in the bronchiolar epithelium, with TGF-β1 also decreased in alveolar macrophages, in patients with stable COPD compared with control smokers with normal lung function. TGF-β3 expression was increased in the bronchial lamina propria of both control smokers with normal lung function and smokers with mild/moderate stable COPD compared with control nonsmokers and correlated significantly with pack-years of smoking. However, TGF-β3^+^ cells decreased in patients with severe/very severe COPD compared with control smokers. Latent TGF-β binding protein 1 expression was increased in the bronchial lamina propria in subjects with stable COPD of all severities compared with control smokers with normal lung function. Bone morphogenetic protein and activin membrane-bound inhibitor expression (BAMBI) in the bronchial mucosa was significantly increased in patients with stable COPD of all severities compared with control subjects. No other significant differences were observed between groups for all the other molecules studied in the bronchial mucosa and peripheral lung.

**Conclusions:**

Expression of TGF-βs and their regulatory proteins is distinct within different lower airway compartments in stable COPD. Selective reduction in TGF-β1 and enhanced BAMBI expression may be associated with the increase in autoimmunity in COPD.

The transforming growth factor-β (TGF-β) family regulates cell proliferation, differentiation, extracellular matrix synthesis, and apoptosis, which are all important processes in COPD pathogenesis. Attenuation of TGF-β signaling leads to pulmonary emphysema in animal models,^1^, ^2^ which may reflect TGF-β1 effects on vascular endothelial growth factor and angiogenesis.^3^, ^4^ TGF-β1 also has a pivotal role in maintaining peripheral tolerance against self-antigens^5^ and controlling autoimmune responses.^6^, ^7^

The TGF-β superfamily has several members: TGF-β exists as three isoforms—TGF-β1, TGF-β2, and TGF-β3—which exhibit similar functions in vitro but may have distinct activities in vivo.^8^ TGF-βs act through specific receptors (TGF-β receptors [TGF-βRs]) I, II, and III. All TGF-βs bind a heteromeric type I/II receptor complex, which activates both “canonical” signals involving SMADs (small mother against decapentaplegic) and “noncanonical” pathways involving mitogen-activated protein kinases (MAPKs) and phosphoinositide 3-kinase (PI3K).^9^

The SMAD family of transcription factors consists of the receptor-regulated SMADs, a common pathway SMAD, and inhibitory SMADs. Receptor-regulated SMADs include SMAD2 and SMAD3, which are recognized by TGF-βRs and activin receptors, and SMADs 1, 5, 8, and 9, which are activated by bone morphogenetic protein (BMP) receptors. SMAD4 or cooperating SMAD is not phosphorylated by the TGF-βRs, whereas inhibitory SMADs (anti-SMADs), including SMAD6 and SMAD7, downregulate TGF-β signaling. SMADs also act as signal integrators and interact with MAPK, nuclear factor-κB, PI3K, and hypoxia-inducible factor 1 signaling pathways.^8^, ^10^

TGF-βRIII (also known as β-glycan) acts as a coreceptor and directly binds TGF-β1, 2, and 3 to enhance their binding to the TGF-βRI/II complex and increase SMAD-dependent signaling.^11^ TGF-β signaling is modulated by connective tissue growth factor (CTGF or CCN2). CNN directly binds TGF-β1 and facilitates binding to the TGF-βRI/II complex enhancing downstream signaling.^12^

The latency-associated peptide (LAP) is associated with latent TGF-β binding proteins (LTBPs) forming a complex known as the large latent complex (LLC).^13^, ^14^ Most TGF-β is secreted as part of the LLC. LTBPs belong to the fibrillin-LTBP family of extracellular matrix (ECM) proteins. LTBP-1 anchors to the ECM and creates traction when LAP binds cell surface integrins; this traction deforms LAP, which releases active TGF-β.^13^, ^15^

Conversely, TGF-βR-associated binding protein (TRAP) 1 inhibits TGF-β1 function by interfering with SMAD3 signaling.^16^ The 5′-TG-3′-interacting factors (TGIFs), TGIF1 and TGIF2, cause repression of TGF-β1-activated genes by direct competition with Smad2.^17^ In addition, BMP and activin membrane-bound inhibitor (BAMBI) acts as a competitive receptor antagonist for TGF-βRI.^18^ BAMBI expression is upregulated by TGF-β1 in a feedback loop.^19^ TGF-β-induced protein (TGFBI, also known as BIG-H3 and keratoepithelin) is an extracellular matrix protein used as a TGF-β1 bioactivity marker.^20^

The aim of this study is to investigate the expression of TGF-β signaling pathways in the lower airways (bronchial mucosa and peripheral lung) of patients with stable COPD and control subjects.

## Methods

### Subjects

All patients with COPD and healthy control subjects were recruited from the Respiratory Medicine Unit of the Istituti Clinici Scientifici Maugeri, Veruno, Italy and the Section of Respiratory Diseases of the University Hospital of Ferrara, Italy. Archival material was used in the present study.^21^ We obtained bronchial biopsy samples from 55 subjects to study immunohistochemically. The characteristics of these subjects are reported in Table 1. Twenty-four subjects undergoing lung resection for a solitary peripheral neoplasm were recruited for the immunohistochemical study of peripheral lung tissue. The characteristics of these subjects are reported in Table 2. COPD and chronic bronchitis were defined according to international guidelines, that is, COPD is the presence of a postbronchodilator FEV_1_/FVC ratio < 70% and chronic bronchitis is the presence of cough and sputum production for at least 3 months in each of two consecutive years according to Global Initiative for Chronic Obstructive Lung Disease criteria (http://www.goldcopd.org). In patients with COPD, the severity of the airflow obstruction was graded using the 2011 GOLD criteria. The GOLD criteria used here to stratify the severity of stable COPD was based on the degree of airflow obstruction, as symptoms and exacerbation rate data were not collected routinely before 2012. Furthermore, the addition of the symptoms and the number of exacerbations are of unproved value in designing studies on the pathogenesis of COPD.

**Table 1:** Clinical Characteristics of Subjects for Immunohistochemical Studies on Bronchial Biopsy Samples

Group	No.	Age (y)	Male/Female	Pack-years of Smoking	Ex/Current Smokers	FEV_1_ (% Predicted) Pre-β_2_	FEV_1_ (% Predicted) Post-β_2_	FEV_1_/FVC (%)
Control nonsmokers	11	67 ± 10	10/1	0	0	116 ± 14	ND	85 ± 10
Control smokers with normal lung function	12	61 ± 7	9/3	43 ± 26	2/10	104 ± 13	ND	81 ± 6
COPD grades I and II (mild/moderate)	14	67 ± 8	12/2	40 ± 19	5/9	66 ± 14^a^	72 ± 12	60 ± 8^a^
COPD grades III and IV (severe/very severe)	18	66 ± 9	11/7	54 ± 36	13/5	35 ± 8^a^^,^^b^	38 ± 9	44 ± 10^a^^,^^b^

Patients with COPD were classified according to Global Initiative for Chronic Obstructive Lung Disease 2011 (http://www.goldcopd.org) grades of severity using only the severity of airflow obstruction. For patients with COPD, FEV_1_/FVC (%) are postbronchodilator values.

ND = not determined; pre-β_2_ = values obtained before bronchodilator use; post-β_2_ = values obtained after bronchodilator use.

Statistical analysis with analysis of variance test:

a *P* < .0001, which was significantly different from control smokers with normal lung function and control never-smokers.

b *P* < .0001, which was significantly different from mild/moderate COPD.

**Table 2:** Characteristics of Subjects for Immunohistochemical Studies on the Peripheral Lung Tissue

Groups	No.	Age (y)	Male/Female	Ex/Current Smokers	Pack-Years of Smoking	Chronic Bronchitis	FEV_1_ (% Predicted)	FEV_1_/FVC (%)
Control smokers	12	63.6 ± 3	10/2	6/6	51.3 ± 11.6	No	87.9 ± 4.5	77.4 ± 1.7
Patients with COPD	12	69.9 ± 1.3	12/0	6/6	45.8 ± 6.1	No	68.6 ± 4.2^a^	58.7 ± 2.5^a^

For COPD and control smoker subjects, FEV_1_ % predicted and FEV_1_/FVC % are postbronchodilator values. Data expressed as mean ± SEM.

a Analysis of variance = *P* < .01.

All patients with COPD were stable, and none of the subjects with COPD were treated with theophylline, antibiotics, antioxidants, mucolytic agents, or glucocorticoids, or any combination thereof, in the month prior to bronchoscopy or lung resection. The study conformed to the Declaration of Helsinki and was approved by the institutional review boards of Istituti Clinici Scientifici Maugeri (protocol p81) and the University Hospital of Ferrara.

### Other Methods

A detailed description of the lung function and fiberoptic bronchoscopy results; the collection, processing, and immunohistochemical analysis of the bronchial biopsy samples and the peripheral lung (e-Tables 1, 2); and the statistical analysis are provided in e-Appendix 1.

## Results

### Clinical Characteristics of Subjects Providing Bronchial Biopsy and Peripheral Lung Samples

We obtained and studied bronchial biopsy samples from 55 subjects. Thirty-two subjects had mild/moderate or severe stable COPD, 12 were current or ex-smokers with normal lung function, and 11 were lifelong nonsmokers with normal lung function (Table 1). In addition, we studied peripheral lung tissue from 24 subjects: 12 smokers with mild/moderate stable COPD and 12 smokers with normal lung function (Table 2).

### Measurement of the Inflammatory Cells in the Bronchial Lamina Propria of COPD and Control Subjects

The data obtained from patients with stable COPD by immunohistochemical analysis confirmed previous results^21^ showing elevated numbers of CD8^+^ T cells, CD68^+^ macrophages, and neutrophils in COPD.

### Immunohistochemical Results for TGF-β Signaling Pathway Members in the Bronchial Epithelium

**Figure 1:**
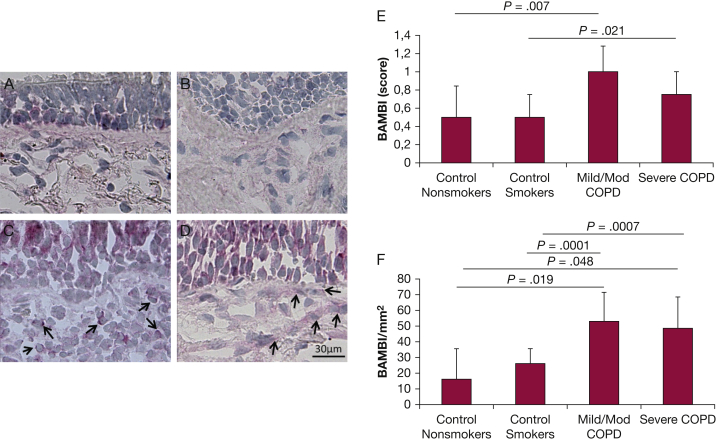
Photomicrographs showing the bronchial mucosa from (A) control nonsmoker, (B) control healthy smoker with normal lung function, (C) mild/moderate stable COPD, (D) severe/very severe stable COPD immunostained for identification of BAMBI^+^ cells (arrows) in the epithelium and bronchial lamina propria. Results are representative of those from 11 nonsmokers, 12 healthy smokers, 14 subjects with mild/moderate COPD, and 18 subjects with severe/very severe COPD. Graphs indicate median (interquartile range) values of (E) BAMBI scored in the epithelium, BAMBI (score), and (F) BAMBI^+^ cells quantified in the lamina propria (BAMBI/mm^2^) of the groups of subjects studied. *P* values were obtained using the Mann-Whitney test for comparison between groups. BAMBI = bone morphogenetic proteins and activin membrane-bound inhibitor.

No differences in the expression of TGF-β1, TGF-β2, TGF-β3, TGF-βRI, TGF-βRII, TGF-βRIII, TGFβ-I/BIGH3, TGIF2, SMAD2, SMAD3, SMAD6, SMAD7, CCN2, LTBP-1, or TRAP-1 were seen in the bronchial epithelia of subjects with COPD compared with nonsmoking control subjects. The number of BAMBI^+^ immunostained cells was significantly increased in the bronchial epithelia of subjects with COPD compared with control subjects (Fig 1A-E, Table 3).

**Table 3:** Immunohistochemical Quantification of TGF-β Signaling Pathways in Bronchial Biopsy Samples

Target	Nonsmokers With Normal Lung Function	Smokers With Normal Lung Function	Mild/Moderate COPD	Severe/Very Severe COPD	Kruskal-Wallis *P* Value
Bronchial epithelium score (0-3)					
TGF-β1	0.25 (0.0-0.75)	0.25 (0.0-0.75)	0.25 (0.0-1.0)	0.37 (0.0-0.75)	.945
TGF-β2	0.12 (0.0-1.5)	0.0 (0.0-1.5)	0.0 (0.0-1.5)	0.25 (0.0-2.0)	.846
TGF-β3	0.0 (0.0-0.0)	0.0 (0.0-0.0)	0.0 (0.0-0.0)	0.0 (0.0-0.0)	n.v.
TGF-βRI	0.0 (0.0-0.25)	0.0 (0.0-0.25)	0.0 (0.0-1.0)	0.0 (0.0-0.25)	.661
TGF-βRII	0.5 (0.2-2.0)	0.5 (0.0-3.0)	0.5 (0.0-2.0)	1.0 (0.0-2.0)	.405
TGFβ-RIII	0.25 (0.0-0.5)	0.25 (0.0-0.75)	0.25 (0.0-1.0)	0.25 (0.0-0.75)	.334
TGFBI/BIGH3	0.0 (0.0-0.0)	0.0 (0.0-0.25)	0.0 (0.0-0.0)	0.0 (0.0-0.25)	.971
TGIF2	1.5 (1.25-2.0)	1.5 (0.75-1.75)	1.75 (0.75-2.5)	1.5 (1.0-2.0)	.185
SMAD2	1.0 (0.0-2.5)	0.5 (0.0-3.0)	0.0 (0.0-2.0)	1.0 (0.0-2.5)	.249
SMAD3	1.0 (0.0-2.0)	0.0 (0.0-3.0)	0.5 (0.0-1.5)	1.0 (0.0-2.5)	.468
SMAD6	0.50 (0.25-1.0)	0.50 (0.0-1.5)	0.75 (0.0-1.5)	0.25 (0.25-1.25)	.296
SMAD7	0.75 (0.0-2.0)	0.5 (0.0-2.5)	0.35 (0.0-2.5)	0.5 (0.0-2.0)	.797
CCN2	1.5 (1.0-1.5)	1.5 (1.0-2.5)	1.75 (1.0-3.0)	1.5 (1.0-2.5)	.866
LTBP-1	0.25 (0.0-0.25)	0.0 (0.0-0.25)	0.25 (0.0-0.75)	0.25 (0.0-0.5)	.263
TRAP-1	0.0 (0.0-0.0)	0.0 (0.0-0.0)	0.0 (0.0-0.0)	0.0 (0.0-0.0)	n.v.
BAMBI	0.5 (0.25-1.25)	0.5 (0.25-0.75)	1.0 (0.25-1.75)^a^	0.75 (0.25-1.5)^b^	.033
Bronchial lamina propria score (cells/mm^2^)					
TGF-β1	27.0 (5.0-60.0)	12.5 (0.0-143.0)	16.0 (0.0-64.0)	28.0 (5.0-141.0)	.752
TGF-β2	28.0 (14.0-69.0)	13.0 (0.0-37.0)	19.5 (0.0-56.0)	23.0 (0.0-68.0)	.211
TGF-β3	0.0 (0.0-13.0)	16.0 (6.0-58.0)^a^	10.0 (0.0-39.0)^a^	4.5 (0.0-65.0)^b^	.007
TGF-βRI	5.0 (0.0-52.0)	6.0 (0.0-75.0)	13.0 (0.0-97.0)	5.0 (0.0-73.0)	.186
TGF-βRII	74.0 (0.0-225.0)	48.0 (0.0-216.0)	72.5 (8.0-505.0)	45.0 (0.0-376.0)	.528
TGF-βRIII	6.0 (4.0-23.0)	6.0 (0.0-32.0)	11.0 (0.0-97.0)	8.0 (5.0-121.0)	.612
TGFBI/BIGH3	251.0 (138-484)	304.0 (174-548)	338.0 (218-408)	361.0 (244-468)	.142
TGIF2	204 (64-352)	169 (77-277)	177 (77-322)	157 (122-235)	.712
SMAD2	181.5 (0.0-750.0)	166.5 (50.0-514.0)	51.0 (0.0-960.0)	91.5 (4.0-627.0)	.530
SMAD3	77.0 (0.0-690.0)	87.0 (6.0-754.0)	111.0 (0.0-909.0)	120.0 (0.0-353,0)	.822
SMAD6	45.5 (12.0-145.0)	60.5 (12.0-148.0)	83.0 (21.0-134.0)	32.0 (9.0-148.0)	.143
SMAD7	67.0 (11.0-300.0)	48.5 (0.0-584.0)	96.0 (0.0-520.0)	56.5 (0.0-620.0)	.949
CCN2	147.0 (84.0-210.0)	86 (52.0-234.0)	111.0 (55.0-312.0)	102.0 (48.0-168.0)	.768
LTBP-1	3.0 (0.0-13.0)	1.5 (0.0-8.0)	5.0 (0.0-65.0)^b^	9.5 (0.0-109.0)^b^	.017
TRAP-1	0.0 (0.0-6.0)	0.0 (0.0-5.0)	0.0 (0.0-12.0)	0.0 (0.0-5.0)	.243
BAMBI	16.0 (7.0-86.0)	26.0 (8.0-35.0)	53.0 (24.0-364.0)^a^^,^^b^	48.5 (16.0-258.0)^a^^,^^b^	.0001

Data are expressed as median (range).

BAMBI = bone morphogenetic protein and activin membrane-bound inhibitor. CTGF = connective tissue growth factor; LTBP-1 = latent transforming growth factor-β1 binding protein 1; n.v. = no value. SMAD = small mother against decapentaplegic TGFBI = transforming growth factor-β-induced protein; TGIF2 = TGF-β-induced factor 2; TRAP-1 = transforming growth factor-β receptor-associated binding protein.

Statistics: The Kruskal-Wallis test was used for multiple comparisons followed by the Mann-Whitney *U* test for comparison between groups.

a *P* < .05, which was significantly different from control nonsmokers.

b *P* < .05, which was significantly different from control smokers.

### Immunohistochemical Results for TGF-β Signaling Pathway Members in the Lamina Propria

No differences in TGF-β1, TGF-β2, TGF-βRI, TGF-βRII, TGF-βRIII, TGFBI/BIGH3, TGIF2, SMAD2, SMAD3, SMAD6, SMAD7, CCN2, or TRAP-1 expression were observed between groups in the lamina propria (Table 3).

The number of TGF-β3^+^ immunostained cells was significantly increased in the bronchial lamina propria of patients with mild/moderate COPD and control smokers with normal lung function compared with nonsmoking control subjects (Table 3). However, subjects with severe/very severe COPD had significantly decreased TGF-β3^+^ cells compared with control smokers with normal lung function. LTBP-1^+^ immunostaining (Table 3) and the number of BAMBI^+^ cells were significantly increased in all severities of COPD compared with control subjects (Fig 1A-F, Table 3). No differences in TGF-β1, TGF-β2, TGF-βRI, TGF-βRII, TGF-βRIII, TGFBI/BIGH3, TGIF2, SMAD2, SMAD3, SMAD6, SMAD7, CCN2, or TRAP-1 expression were observed between groups in the lamina propria.

### Immunohistochemical Results of the TGF-β Signaling Pathway in the Peripheral Lung

**Figure 2:**
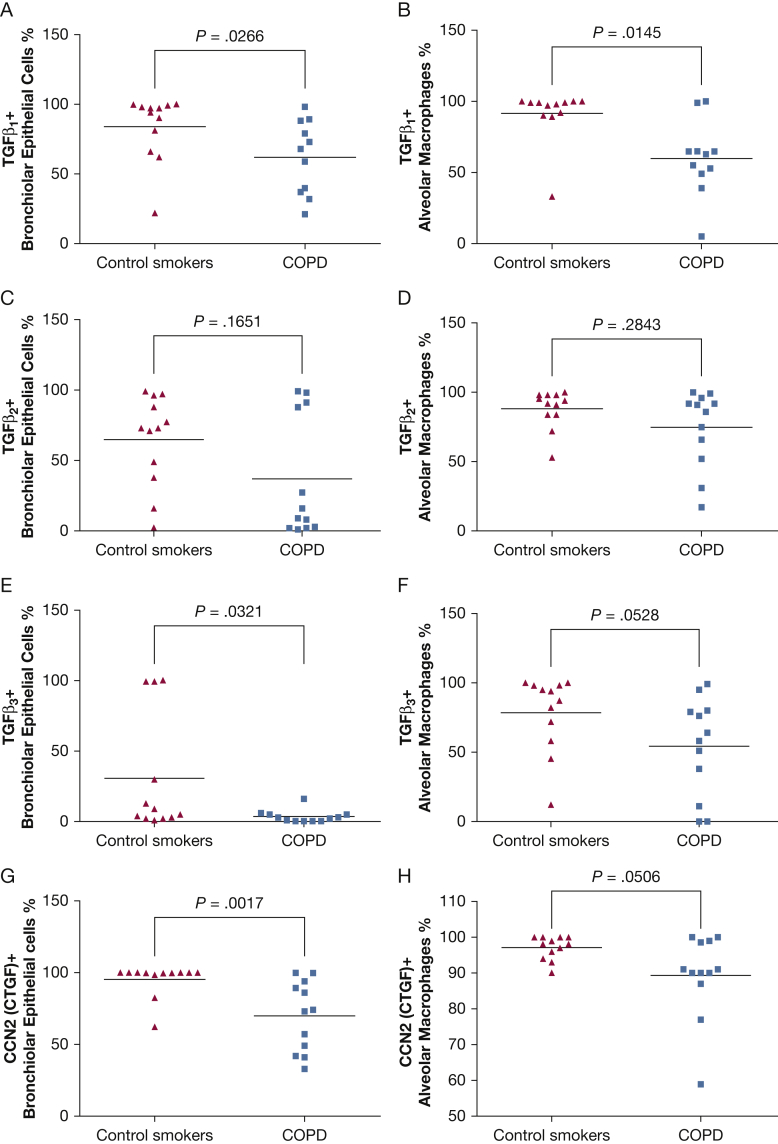
The percentage of the (A) bronchiolar epithelial and (B) alveolar macrophage cells immunostained for TGF-β1 and (G and H) CCN2 (CTGF). Values for (C and D) TGF-β2 and (E and F) TGF-β3 are shown. Results are representative of those from 12 subjects with stable COPD and 12 control smokers with normal lung function. The Mann-Whitney U test was used for statistical analysis. Exact *P* values are shown above each graph.

At variance with the bronchial epithelium, the percentage of TGF-β1^+^ (Fig 2A, e-Fig 1), TGF-β3^+^ (Fig 2E, e-Fig 2), and CCN2^+^ (Fig 2G, e-Fig 3) bronchiolar epithelial cells in the peripheral airways was significantly decreased in patients with COPD compared with control smokers with normal lung function (Table 4). The percentage of TGF-β1^+^ alveolar macrophages was decreased in patients with COPD compared with control smokers with normal lung function (Fig 2B, Table 4). There was also a trend for decreased TGF-β3^+^ and CCN2^+^ immunostained alveolar macrophages in patients with COPD (Figs 2F, 2H, Table 4). No other significant differences were observed between groups for any other molecules studied (Fig 2, Table 4, e-Fig 4).

**Table 4:** Immunohistochemical Quantification of TGF-β Signaling Pathways in the Peripheral Lung

Localization and Antigen	Control Smokers	COPD	Mann-Whitney *U* Test *P* Value
Bronchiolar epithelium (cells, percentage)			
TGF-β1	95.5 (69.8-98.8)	68.0 (37.0-88.0)	.0266
TGF-β2	73.0 (40.8-94.0)	12.5 (2.3-90.3)	.1651
TGF-β3	7.0 (2.3-81.8)	2.5 (0.4-5.0)	.0321
TGF-β-RI	85.0 (80.0-92.3)	91.5 (84.0-96.5)	.1081
TGF-β-RII (bronchiolar smooth muscle)	15.5 (8.3-23.5)	11.0 (0.5-20.3)	.4003
TGF-βRIII	0.0 (0.0-0.0)	0.0 (0.0-0.0)	NA
TGIF2 (nuclear)	3.0 (0.0-21.0)	12.5 (7.5-19.8)	.1623
TGIF2 (apical)	60.0 (34.5-85.3)	52.5 (45.5-73.0)	.9539
SMAD2 (nuclear)	9.0 (4.0-21.5)	6.0 (1.3-34.8)	.7947
SMAD2 (cytosolic)	10.5 (1.5-22.5)	1.0 (0.0-7.8)	.0610
SMAD3	5.0 (2.3-30.8)	15.5 (1.0-45.5)	.7066
SMAD6	100.0 (99.8-100.0)	100.0 (99.2-100.0)	.6148
SMAD7	99.0 (97.3-99.7)	99.0 (93.0-99.0)	.5516
TRAP-1 (nuclear)	5.0 (0.3-12.8)	6.0 (0.3-15.5)	.8612
TRAP-1 (cytosolic)	0.0 (0.0-0.0)	0.0 (0.0-7.5)	.4255
CCN2	100.0 (98.8-100.0)	73.5 (43.8-92.8)	.0017
LTBP-1	0.0 (0.0-0.0)	0.0 (0.0-0.0)	.8939
BAMBI (nuclear)	55.5 (38.2-75.1)	51.3 (40.0-60.3)	.5833
BAMBI (cytosolic)	4.0 (2.1-6.6)	3.5 (1.2-5.8)	.5031
Alveolar macrophages (cells, percentage)			
TGF-β1	98.5 (90.5-99.8)	63.0 (50.0-66.0)	.0145
TGF-β2	93.0 (84.0-98.0)	88.5 (55.5-95.0)	.2843
TGF-β3	90.5 (61.5-98.0)	61.0 (17.8-79.8)	.0528
TGF-βRI	87.5 (34.0-93.5)	85.0 (70.8-97.8)	.5435
TGF-βRII	11.0 (0.0-40.5)	29.5 (12.0-47.8)	.1737
TGF-βRIII	18.0 (11.0-63.8)	42.5 (20.8-76.0)	.2850
TGIF2 (nuclear)	7.5 (2.3-23.0)	7.5 (1.3-15.8)	.7501
TGIF2 (cytosolic)	73.5 (45.3-93.3)	73.5 (72.3-81.0)	.9769
SMAD2 (nuclear)	0.0 (0.0-0.0)	0.0 (0.0-1.8)	.3496
SMAD2 (cytosolic)	63.0 (41.0-82.0)	60.5 (39.3-85.8)	.9770
SMAD3 (nuclear)	0.0 (0.0-1.0)	0.0 (0.0-1.0)	.9175
SMAD3 (cytosolic)	39.5 (32.0-68.3)	27.0 (10.0-79.8)	.4024
SMAD6	100.0 (100.0-100.0)	100.0 (100.0-100.0)	NA
SMAD7	100.0 (100.0-100.0)	100.0 (100.0-100.0)	.3384
TRAP-1 (nuclear)	14.0 (11.0-19.0)	22.5 (1.8-24.0)	.1558
TRAP-1 (cytosolic)	79.0 (65.0-82.8)	76.5 (63.0-79.5)	.3545
CCN2	98.0 (94.5-100.0)	90.5 (87.8-98.9)	.0506
LTBP-1	29.5 (15.8-46.5)	45.0 (29.5-77.3)	.1120
BAMBI (nuclear)	53.8 (7.0-66.8)	18.5 (10.8-31.1)	.0832
BAMBI (cytosolic)	32.5 (24.2-40.0)	42.5 (26.0-59.5)	.2038
Lung vessels (score)			
TGFBI/BIGH3	1.0 (1.0-1.8)	1.0 (1.0-2.0)	.3132
SMAD2	1.0 (1.0-2.0)	1.5 (1.0-2.8)	.3440

Data expressed as median (range).

Statistics: The Mann-Whitney *U* test was applied for comparison between groups. Note: when not specified, the positive staining is intended to be nuclear, apart from TGFβ-RI, in which it is apical in the bronchiolar epithelium and cytosolic in the alveolar macrophages, and TGF-βRIII and LTBP-1, for which it is cytosolic.

NA = not applicable. See Table 3 legend for expansion of other abbreviations.

### Analysis of Gene Expression Data in Large and Small Airway Epithelial Cells

We examined the relative expression of TGF-β1, TGF-β2, TGF-β3, CCN2, LTBP1, and BAMBI mRNA in epithelial cells from the small (GSE11784) and large airways (GSE37147) of patients with COPD compared with control subjects (e-Table 3) using previously published data sets. These data demonstrated a lack of concordance between protein and mRNA for TGF-β pathway members (compare results in e-Table 4 with those in Tables 3 and 4). BAMBI mRNA expression was not different between bronchial epithelial cells from subjects with COPD (n = 30) and healthy smokers (n = 69), although active smoking had a small significant (adjusted *P* = .03) 1.14-fold increase in BAMBI mRNA (e-Table 3). In contrast, LTBP-1 mRNA expression in subjects with COPD was significantly less than that in nonsmokers (adjusted *P* = 8.74 × 10^–14^).

In contrast to the reduction in TGF-β1, TGF-β3, and CCN2 protein expression in COPD compared with cells from healthy smokers, there was no difference in TGF-β3 mRNA in small airway epithelial cells, but there was an increase in TGF-β1 and CCN2 mRNA expression (e-Table 3). TGF-β1 mRNA expression was significantly increased at 1.22-fold (adjusted *P* = .00194) and CCN2 was increased by 1.3-fold (adjusted *P* = .0024) in cells from subjects with COPD (n = 36) vs those from healthy nonsmokers (n = 69).

### Correlations Between Clinical Parameters, Inflammatory Cell Counts, and TGF-β Signaling Pathway in Bronchial Biopsy Samples

**Figure 3:**
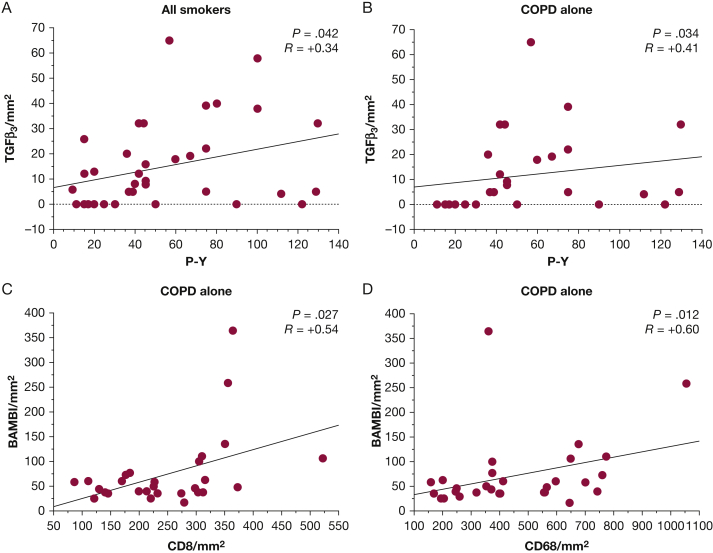
Regression analysis between pack-years and number of TGF-β3^+^ cells infiltrating the bronchial lamina propria in (A) all smokers (with and without COPD) and (B) patients with COPD alone. In the latter patients, there is a significant positive correlation between the number of BAMBI^+^ cells in the bronchial lamina propria and those of (C) CD8^+^ and (D) CD68^+^ cells. Correlation coefficients were calculated by using the Spearman rank method. See Figure 1 legend for expansion of abbreviations.

There was a significant correlation between the number of cigarettes smoked (pack-years) and the number of TGF-β3^+^ immunostained cells/mm^2^ in the bronchial lamina propria when subjects with or without COPD were grouped together (Fig 3A). This correlation was maintained within the COPD group alone (Fig 3B). The numbers of BAMBI^+^ immunostained cells/mm^2^ in the bronchial lamina propria were significantly correlated with numbers of CD8^+^ cells/mm^2^ (Fig 3C) and CD68^+^ cells/mm^2^ (Figure 3D). No other significant correlations were observed between groups for all the other molecules studied.

## Discussion

We report here for the first time, to our knowledge, the comprehensive expression and localization of TGF-β regulatory proteins in the lower airways of patients with stable COPD compared with control subjects. We observed decreased expression of TGF-β1 and TGF-β3 in the bronchiolar but not bronchial epithelium and of TGF-β1 in alveolar macrophages of patients with stable COPD compared with control smokers with normal lung function. Furthermore, TGF-β3 expression was increased in the bronchial lamina propria of control smokers with normal lung function and mild/moderate stable COPD compared with control nonsmokers and correlated significantly with pack-years. These data suggest that the TGF-β signaling is selectively impaired in the small airway epithelia of patients with stable COPD. The expression of the TGF-β pseudoreceptor BAMBI was also elevated in the bronchial mucosa of patients with COPD.

Conflicting results have previously been reported regarding the expression of the TGF-β signaling pathway in patients with stable COPD in bronchial biopsy samples and peripheral lung (e-Table 4). Our data for TGF-β1 agree with some previous studies^22^, ^23^ but not others.^24^, ^25^ The latter studies found increased TGF-β1 expression in the small airway and alveolar epithelial cells, but when the control subjects used for the study are classified according to current GOLD criteria using FEV_1_/FVC ratios, many would be reclassified as having COPD.^24^ Another study reported increased TGF-β1 protein for both COPD and control smokers compared with nonsmoking subjects.^26^ A more recent study found increased release of total TGF-β1 from bronchial epithelium in vitro obtained from patients with COPD compared with control subjects without any significant differences in active TGF-β1 release.^27^ Finally, another study found no significant differences in BAL TGF-β1 levels between patients with COPD and control subjects.^28^ These apparent discrepancies may be explained by incomplete clinical features or the selectivity of the primary anti-TGF-β1 antibodies used, or both.^29^, ^30^

Llinàs et al^31^ observed decreased TGF-β1 mRNA expression in the peripheral lungs of patients with severe stable COPD compared with control nonsmoking subjects, and in agreement with our present data, Kokturk et al^32^ found no difference in TGF-β1 immunohistochemical expression in the bronchial biopsy samples from patients with stable COPD and control nonsmoking subjects. Vignola et al^33^ demonstrated increased TGF-β1 immunostaining in the bronchial biopsy samples of patients with chronic bronchitis compared with control young nonsmoking subjects (mean age, 46 years). However, these patients had a mean FEV_1_ of 56% to 90% predicted, but only nine subjects were considered to have COPD using unspecified criteria.^33^ Hence, these differences render the comparison of our results with the previous studies difficult.^33^, ^34^ Decreased expression of TGF-β1 in the small airways in COPD shown here and its critical role in regulating self-tolerance^5^ may explain the autoimmunity seen in some patients with COPD.^6^, ^7^ The complex signaling control points within the TGF-β activation pathway may enable targeted treatment of this complication.

TGF-β3 expression was increased in the bronchial lamina propria of patients with COPD and control smokers compared with control nonsmoking subjects, although TGF-β3^+^ cells were decreased in patients with severe/very severe COPD compared with control smokers. There was a significant correlation between pack-years and the number of TGF-β3^+^ immunostained cells/mm^2^ in the bronchial lamina propria. To our knowledge, this is the first report of TGF-β3 protein expression and localization in the lower airways of patients with stable COPD and is in keeping with the results of decreased TGF-β3 mRNA expression in the peripheral lung of patients with more severe COPD^31^, ^35^ and of increased TGF-β3 mRNA expression in the peripheral lung of smokers compared with nonsmokers.^31^ This concordance was not observed with our present mRNA results.

TGF-β3 protein release from bronchial epithelium in vitro and BAL TGF-β3 levels were similar between patients with COPD and control subjects.^27^, ^28^ We were unable to find any significant differences between patients with stable COPD and control subjects in TGF-β2 and TGF-βR expression and localization in the lower airways. In addition, we could not confirm the decreased TGF-βRI protein expression observed in the peripheral lungs of patients with moderate stable COPD compared with control subjects in previous studies.^23^, ^31^ This discrepancy may be explained by differences in anti-TGF-βRI antibody used. In contrast, our results on TGF-βRII protein expression are in agreement with the data from the same study^23^and with the mRNA data from Llinàs et al.^31^

To our knowledge, we have provided the first data on TGF-βRIII protein expression, showing its low immune expression in the lower airways of patients with stable COPD and control subjects. Single nucleotide polymorphisms are likely to be associated with decreased TGF-βRIII function and are linked to an increased risk of pulmonary emphysema developing.^36^ This area requires further research.

We found no significant differences between patients with stable mild/moderate COPD and control subjects (including smokers with normal lung function) in SMAD2, SMAD3, SMAD6, and SMAD7 expression and localization in the lower airways, confirming previous studies.^23^, ^37^, ^38^ In contrast, reduced SMAD6 and SMAD7 mRNA expression was reported in bronchial biopsy samples from patients with stable COPD,^39^ as well as decreased SMAD7 mRNA in the bronchial epithelium^40^ and decreased SMAD mRNA expression^41^ and SMAD protein expression in the peripheral lung.^42^ These discrepancies may be explained by the lack of concordance between mRNA and protein, as we described in our study comparing gene expression data, or by the potential different pathogenic pathways behind the onset of COPD vs pulmonary emphysema.^43^, ^44^, ^45^ Overall, these data suggest limited involvement of SMAD signaling in the pathogenesis of lower airway inflammation and damage in patients with stable mild/moderate COPD.

We also demonstrated decreased CCN2 (CTGF) expression in the bronchiolar epithelium but not in the bronchial mucosa of patients with stable COPD compared with control smokers with normal lung function. Conflicting results have previously been reported for CCN2 mRNA expression in patients with COPD.^31^, ^46^ TGF-β1 induces CCN2 expression in fibroblasts in vitro,^47^ suggesting that the decreased expression of both TGF-β1 and CCN2 observed in the bronchiolar epithelium of the patients in our study with stable COPD may be related. Lung specimens from subjects with a solitary peripheral neoplasm were used in the present study, raising the question as to whether lung tumors may influence the results. The presence of similar pathologic conditions in smokers and patients with COPD and the large number of studies already published examining inflammatory markers and cytokine pathways in similar sets of subjects support the use of these groups of patients for peripheral lung studies.

LTBP-1 is the only LTBP that both interacts with latent TGF-βs^48^ and is predominantly expressed in the lungs.^49^, ^50^, ^51^, ^52^, ^53^ LTBP-1 immunostaining was increased in the bronchial lamina propria of patients with COPD compared with control smokers with normal lung function. LTBP-1 may activate TGF-β3, but the concomitant upregulation of TGF-β3 observed in the bronchial lamina propria in our study is smoking related and not disease related, suggesting the presence of alternative mechanisms regulating the expression of these two molecules. In fact, a substantial amount of LTBP-1 can be secreted by cells without being bound to latent TGF-βs, and TGF-β-independent functions for LTBP-1 need to be determined.^54^

We observed a marked increase in the expression of the TGF-β pseudoreceptor BAMBI in the bronchial mucosa but not in the peripheral airways of patients with stable COPD compared with control subjects in keeping with previous studies in COPD peripheral lung.^55^ There was a positive correlation between the number of BAMBI^+^ and CD8^+^ and CD68^+^ cells. These data are in keeping with the enhanced plasma BAMBI levels recently described in stable COPD that positively correlated with the blood Th17/regulatory T cells (Treg) ratio.^56^ In a mouse model of autoimmune arthritis, BAMBI deficiency protected mice against the development of disease by modulating Th17/Treg differentiation.^57^ This may account for the autoimmune and the Th17/Treg imbalance that we and others have previously described in the bronchial mucosa of patients with stable COPD.^6^, ^7^, ^58^

The mechanisms regulating BAMBI expression are poorly understood. In vitro, BAMBI expression can be upregulated by TGF-β1.^19^ However, the discrepancy observed between TGF-β1 and BAMBI expression in the different compartments of the lower airways suggest that non-TGF-β1-dependent pathways could be involved in BAMBI upregulation in stable COPD. As a potential limitation of this study, we did not apply multiple corrections in the statistical analysis of differences between groups for our “ex vivo” data of the lower airways. In fact, applying multiple corrections can lead to false-negative findings, but by not applying them, findings might be false positive. We were confident that applying a multiple test (analysis of variance or Kruskal-Wallis) followed by a “restricted” test analyzing differences between groups could be sufficiently stringent for identification of true differences.

In conclusion, the reported differences in TGF-β1 and BAMBI expression may contribute to the pathogenesis of stable COPD creating a microenvironment facilitating local autoimmune responses associated with COPD.

## Supplementary Data

e-Online Data
