# Supplementary material for: TGF-β Signaling Pathways in Different Compartments of the Lower Airways of Patients With Stable COPD
Source: Chest. 2018 Apr;153(4):851–62. doi: 10.1016/j.chest.2017.12.017 (PMC5883327; doi:10.1016/j.chest.2017.12.017)
Supplement: e-Online Data [file mmc1.pdf]

CHEST Online Supplement

# TGF-β Signaling Pathways in Different Compartments of the Lower Airways of Patients With Stable COPD

*Antonino Di Stefano, PhD; Claudia Sangiorgi, PhD; Isabella Gnemmi, PhD; Paolo Casolari, PhD; Paola Brun, PhD; Fabio L. M. Ricciardolo, MD; Marco Contoli, MD; Alberto Papi, MD; Pio Maniscalco, MD; Paolo Ruggeri, MD; Giuseppe Girbino, MD; Francesco Cappello, MD; Stelios Pavlides, PhD; Yike Guo, PhD; Kian Fan Chung, MD; Peter J. Barnes, MD; Ian M. Adcock, PhD; Bruno Balbi, MD; and Gaetano Caramori, MD*

CHEST 2018; 153(4):851-862

*Online supplements are not copyedited prior to posting and the author(s) take full responsibility for the accuracy of all data.*

**© 2018 AMERICAN COLLEGE OF CHEST PHYSICIANS.** Reproduction of this article is prohibited without written permission from the American College of Chest Physicians. See online for more details. **DOI:** j.chest.2017.12.017

**e-Appendix 1.**

**Methods**

***Lung function tests and volumes***

Pulmonary function tests were performed as previously described (**S1,S2**) according to published guidelines (**S3**). Pulmonary function tests included measurements of $FEV_1$ and $FEV_1$/FVC under baseline conditions in all the subjects examined (6200 Autobox Pulmonary Function Laboratory; Sensormedics Corp., Yorba Linda, CA). In order to assess the reversibility of airflow obstruction and post bronchodilator functional values the $FEV_1$ and $FEV_1$/FVC% measurements in the groups of subjects with $FEV_1/FVC\% \leq 70\%$ pre-bronchodilator was repeated 20 min after the inhalation of 0.4 mg of salbutamol.

***Fiberoptic Bronchoscopy, Collection and Processing of Bronchial Biopsies***

Subjects were at the bronchoscopy suite at 8.30 AM after having fasted from midnight and were pre-treated with atropine (0.6 mg IV) and midazolam (5-10 mg IV). Oxygen (3 l/min) was administered via nasal prongs throughout the procedure and oxygen saturation was monitored with a digital oximeter. Using local anesthesia with lidocaine (4%) to the upper airways and larynx, a fiberoptic bronchoscope (Olympus BF10 Key-Med, Southend, UK) was passed through the nasal passages into the trachea. Further lidocaine (2%) was sprayed into the lower airways, and four bronchial biopsy specimens were taken from segmental and subsegmental airways of the right lower and upper lobes using size 19 cupped forceps. Bronchial biopsies for immunohistochemistry were gently extracted from the forceps and processed for light microscopy as previously described (**S3**). At least two samples were embedded in Tissue Tek II OCT (Miles Scientific, Naperville, IL), frozen within 15 min in isopentane pre-cooled in liquid nitrogen, and stored at –80°C. The best frozen sample was then oriented and 6μm thick cryostat sections were cut for immunohistochemical light microscopy analysis and processed as described below.

***Collection and Processing of the Peripheral Lung Tissue***

Twenty four subjects undergoing lung resection surgery for a solitary peripheral neoplasm were recruited. Twelve were smokers with normal lung function and 12 subjects were smokers with COPD (table 2 of the main manuscript). All former smokers had stopped smoking for more than one year. All subjects did not undergo preoperative chemotherapy and/or radiotherapy and had not been treated with bronchodilators, theophylline, antibiotics, antioxidants and/or glucocorticoids in the month prior to surgery. Lung tissue processing was performed as previously described (**S4,S5**). Two to four randomly selected tissue blocks were taken from the subpleural parenchyma of the lobe obtained at surgery,

*Online supplements are not copyedited prior to posting and the author(s) take full responsibility for the accuracy of all data.*

avoiding areas grossly invaded by tumour. Samples were fixed in 4% formaldehyde in phosphate-buffered saline at pH 7.2 and, after dehydration, embedded in paraffin wax. Serial sections 4 µm thick were first cut and stained with haematoxylin-eosin (H&E) in order to visualize the morphology and to exclude the presence of microscopically evident tumour infiltration. Tissue specimens were then cut for immunohistochemical analysis and were placed on charged slides as previously reported (**S5**).

***Immunohistochemistry on OCT-Embedded Bronchial Biopsies***

One section from each sample was stained applying immunohistochemical methods with a panel of antibodies specific for inflammatory cells or TGFβ related cytokines and proteins (e-**table 1**). Briefly, after blocking non-specific binding sites with serum derived from the same animal species as the secondary antibody, primary antibody was applied at optimal dilutions in TRIS-buffered saline (0.15 M saline containing 0.05 M TRIS-hydrochloric acid at pH 7.6) and incubated 1hr at room temperature in a humid chamber. Antibody binding was demonstrated with secondary anti-mouse (Vector, BA 2000), anti-rabbit (Vector, BA 1000) or anti-goat (Vector, BA 5000) antibodies followed by ABC kit AP AK5000, Vectastain and fast-red substrate (red color) or ABC kit HRP Elite, PK6100, Vectastain and diaminobenzidine substrate (brown color). Human tonsil or nasal polyp were used as positive controls. For the negative control, normal goat (sc—2048), mouse (sc-2025) or rabbit (sc-2027) non-specific immunoglobulins (Santa Cruz Biotechnology, Santa Cruz, CA, USA) were used at the same protein concentration as the primary antibody.

***Immunohistochemistry in Human Peripheral Lung Tissue***

Immunostaining of paraffin embedded peripheral lung tissue was performed as previously described (**S5**). After deparaffinization and rehydration to expose the immunoreactive epitopes, the sections to be stained, immersed in retrieval solution citrate pH 6.0 or EDTA pH 8.0 were incubated in a microwave oven (model NN S200W; Panasonic, Milano, Italy) on high power for 40 min. Endogenous peroxidase activity was blocked by incubating slides in 3% hydrogen peroxide ($H_2O_2$) in phosphate-buffered saline (PBS) followed by washing in PBS. Cell membranes were permeabilised adding 0.1% saponin to the PBS. Non-specific labeling was blocked by coating with blocking serum (5% normal goat serum) for 20 minutes at room temperature. After washing in PBS the sections were incubated with the following primary antibodies (e-**table 2**) in the experimental conditions reported (e-**table 2**). For the negative control slides normal rabbit, goat or mouse non-specific immunoglobulins (Santa Cruz Biotechnology) were used at the same protein concentration as the primary antibody. Control slides were included in each staining run using human

Online supplements are not copyedited prior to posting and the author(s) take full responsibility for the accuracy of all data.

normal tonsils (kindly provided by Prof Stefano Pelucchi, ENT Section at the University Hospital of Ferrara, Italy) as a positive control for all the immunostaining performed. After repeated washing steps with PBS, the sections were subsequently incubated with goat anti-rabbit, horse anti-mouse or rabbit anti goat biotinylated antibody (Vector ABC Kit, Vector Laboratories; www.vectorlabs.com) for 30 minutes at room temperature. After further washing the sections were subsequently incubated with ABC reagent (Vector ABC Kit, Vector Laboratories) for 30 minutes at room temperature. Slides were then incubated with chromogen-fast diaminobenzidine (DAB) as chromogenic substance. After which they were counterstained in haematoxylin and mounted on permanent mounting medium.

***Scoring System for Immunohistochemistry in the Bronchial Biopsies***

Morphometric measurements were performed with a light microscope (Leitz Biomed, Leica Cambridge, UK) connected to a video recorder linked to a computerized image system (Quantimet 500 Image Processing and Analysis System, Software Qwin V0200B, Leica). Light-microscopic analysis was performed at a magnification of 630x.

The immunostaining for all the antigens studied was scored (range: 0 = absence of immunostaining to 3 = extensive intense immunostaining) in the intact (columnar and basal epithelial cells) bronchial epithelium, as previously described (S3). The final result was expressed as the average of all scored fields performed in each biopsy. A mean±SD of 0.700±0.260 millimeters of epithelium was analyzed in COPD patients and control subjects.

Immunostained cells in the bronchial lamina propria were quantified 100μm beneath the epithelial basement membrane in several non-overlapping high-power fields until the whole specimen was examined. The final result, expressed as the number of positive cells per square millimeter, was calculated as the average of all the cellular counts performed in each biopsy.

***Scoring System for Immunohistochemistry in the Peripheral Lung Tissue***

Staining analysis was performed as previously published (**S3, S5**). Staining data were interpreted blinded with no prior knowledge of the clinic-pathologic parameters. A bronchiole was taken to be an airway with no cartilage and glands in its wall. To quantify TGFβ related molecules (TGFβ1, TGFβ2, TGFβ3, TGFβRI, TGFβRII, TGFβRIII, CTGF (CCN2), SMAD2, SMAD3, SMAD6, SMAD7, LTBP-1, BAMBI, TRAP-1, TGIF2 and TGFBI) the area of bronchiolar epithelium to be studied was selected randomly. Cells with nuclear immunostaining were counted on each of 10 consecutive, non-overlapping, high power fields (about 300 cells) with one count on each of three, when available, bronchioles for each section stained. Results were expressed as percentages of total bronchiolar epithelial

Online supplements are not copyedited prior to posting and the author(s) take full responsibility for the accuracy of all data.

cells counted. TGFβRII was expressed and quantified only in the bronchial smooth muscle cells; TGFBI/BIGH3 was only expressed and quantified in the lung vessels.

To quantify TGFβ related molecules (TGFβ1, TGFβ2, TGFβ3, TGFβRI, TGFβRII, TGFβRIII, CTGF (CCN2), SMAD2, SMAD3, SMAD6, SMAD7, LTBP-1, BAMBI, TRAP-1, TGIF2 and TGFBI) expression in alveolar macrophages, at least 20 high-power fields (hpf) of lung parenchyma were randomly selected for each section and at least 100 macrophages inside alveoli were evaluated. Alveolar macrophages were defined as mononuclear cells with well represented cytoplasm present in the alveolar spaces and not attached to the alveolar walls using a previously validated method (**S3, S5)**). Results were expressed as percentages of total alveolar macrophages counted.

**Analysis of differential gene expression**

Quality controlled gene expression data from the small and large airways of COPD patients and controls, normalised with the Robust Multi-array Average (RMA) method, was obtained from Gene Expression Omnibus (GSE11784 and GSE37147 for small and large airways respectively). Differential gene expression analysis was performed with a regression based method, using the Linear models for Microarray Analysis R limma library.

***Statistical analysis***

Group data were expressed as mean (standard deviation) for functional data or median (range) or interquartile range (IQR) for morphologic data. Differences between groups were analyzed using analysis of variance (ANOVA) for functional data. The ANOVA test was followed by the unpaired t-test for comparison between groups. The Kruskal Wallis test applied for morphologic data was followed by the Mann-Whitney U test for comparison between groups. Multiple corrections were not applied in the "ex-vivo" statistical analysis of the data to overcome the issue of false negatives. *In vitro* data were analyzed by the Mann-Whitney U test. Correlation coefficients were calculated using the Spearman rank method. Probability values of $p<0.05$ were considered significant. Data analysis was performed using the Stat View SE Graphics program (Abacus Concepts Inc., Berkeley, CA-USA).

*Online supplements are not copyedited prior to posting and the author(s) take full responsibility for the accuracy of all data.*

**e-Table 1. Primary antibodies and immunohistochemical conditions used for identification of TGFβ signaling-pathways components in bronchial biopsies**

| Target | Supplier | Cat.#[a] | Source(Isotype) | Dilution | Positive control |
|---|---|---|---|---|---|
| **TGF β1** | R&D | MAB 240 | Mouse (IgG1) | 1:20 (20μg/ml) | Nasal polyp |
| **TGF β2** | R&D | MAB 612 | Mouse (IgG2b) | 1:30 (16μg/ml) | Nasal polyp |
| **TGF β3** | R&D | MAB 643 | Mouse (IgG1) | 1:50 (10μg/ml) | Nasal polyp |
| **TGF βRI** | Lab Vision | RB-10455 | Rabbit (IgG) | 1:40 (5μg/ml) | Nasal polyp |
| **TGF βRII** | Santa Cruz | Sc-220 | Rabbit (IgG) | 1:100 (2μg/ml) | Nasal polyp |
| **TGF βRIII** | Bioscience | E11234 | Rabbit (IgG) | 1:25 (10μg/ml) | Nasal polyp |
| **TGFBI/BIGH3** | Proteintech | 60007-1-Ig | Mouse (IgG2a) | 1:150 (10μg/ ml) | Nasal polyp |
| **TGIF2** | Abcam | AB-190152 | Rabbit (IgG) | 1:80 (5μg/ml) | Nasal polyp |
| **SMAD2** | Zymed | 511300 | Rabbit (IgG) | 1:25 (10μg/ml) | Nasal polyp |
| **SMAD3** | Zymed | 511500 | Rabbit (IgG) | 1:25 (10μg/ml) | Nasal polyp |
| **SMAD6** | Abnova | H00004091 | Mouse (IgG2b) | 1:500 (2μg/ml) | Nasal polyp |
| **SMAD7** | Santa Cruz | Sc-11392 | Rabbit (IgG) | 1:25 (4μg/ml) | Nasal polyp |
| **CCN2** | Santa Cruz | Sc-14939 | Goat (IgG) | 1:75 (3μg/ml) | Nasal polyp |
| **LTBP-1** | Santa Cruz | Sc-28132 | Goat (IgG) | 1:50 (4μg/ml) | Nasal polyp |
| **TRAP-1** | Santa Cruz | Sc-13134 | Mouse (IgG2b) | 1:40 (5μg/ml) | Nasal polyp |
| **BAMBI** | Thermo Scientific | PA5-38027 | Rabbit (IgG) | 1:200 (5μg/ml) | Nasal polyp |

[a]Cat#, catalogue number

*Online supplements are not copyedited prior to posting and the author(s) take full responsibility for the accuracy of all data.*

**e-Table 2.** Primary antibodies and immunohistochemical conditions used for identification of TGF-beta signaling pathway components in the peripheral lung

| Primary antibody specificity | Company manufacturer | Catalogue number | Source/ Host | Concentration | Biotinylated secondary antibody | Chromogen |
|---|---|---|---|---|---|---|
| TGF-β1 | R&D | MAB240 | Mouse | 4 µg/ml | LSAB+ kit, DAKO K0690 | DAB |
| TGF-β2 | R&D | MAB612 | Mouse | 5 µg/ml | LSAB+ kit, DAKO K0690 | DAB |
| TGF-β3 | R&D | MAB643 | Mouse | 4 µg/ml | LSAB+ kit, DAKO K0690 | DAB |
| TGFβ-RI | Abcam | ab31013 | Rabbit | 0.4 µg/ml | Biotinylated goat anti-rabbit IgG, Vector (BA 1000); 1:200 | DAB |
| TGFβ-RII | Santa Cruz | sc-400 | Rabbit | 4 µg/ml | Biotinylated goat anti-rabbit IgG, Vector (BA 1000); 1:200 | DAB |
| TGFβ-RIII | Novus Biologicals | NBP1-89988 | Rabbit | 1 µg/ml | Biotinylated goat anti-rabbit IgG, Vector (BA 1000); 1:200 | DAB |
| TGFBI/BIGH3 | Proteintech | 60007-1-lg | Mouse | 0.262 µg/ml | Biotinylated horse anti-mouse IgG, Vector (BA 2000); 1:200 | DAB |
| TGFβ-induced factor 2 C-terminal | Abcam | ab190152 | Rabbit | 2.5 µg/ml | Biotinylated goat anti-rabbit IgG, Vector (BA 1000); 1:200 | DAB |
| TRAP-1 | Santa Cruz | sc-13134 | Mouse | 8 µg/ml | Biotinylated horse anti-mouse IgG, Vector (BA 2000); 1:200 | DAB |
| LTBP-1 | Santa Cruz | sc-28132 | Goat | 2 µg/ml | Biotinylated rabbit anti-goat IgG, Vector (BA 5000); 1:200 | DAB |
| CCN2 | Santa Cruz | sc-14939 | Goat | 4 µg/ml | Biotinylated rabbit anti-goat IgG, Vector (BA 5000); 1:200 | DAB |
| BAMBI | Thermo Scientific | PA5-38027 | Rabbit | 0.71 µg/ml | Biotinylated goat anti-rabbit IgG, Vector (BA 1000); 1:200 | DAB |
| SMAD2 | Santa Cruz | sc-393312 | Mouse | 1 µg/ml | Biotinylated horse anti-mouse IgG, Vector (BA 2000); 1:200 | DAB |
| SMAD3 | Atlas | HPA067203 | Rabbit | 0.5 µg/ml | Biotinylated goat anti-rabbit IgG, Vector (BA 1000); 1:200 | DAB |
| SMAD6 | Santa Cruz | sc-26401 | Goat | 4 µg/ml | LSAB+ kit, DAKO K0690 | DAB |
| SMAD7 | Santa Cruz | sc-101152 | Mouse | 4 µg/ml | LSAB+ kit, DAKO K0690 | DAB |

*Online supplements are not copyedited prior to posting and the author(s) take full responsibility for the accuracy of all data.*

**e-Table 3. Comparison of the gene expression of TGFβ signalling pathway members in small and large airway epithelial cells**

**Small airways GSE11784**

| COPD – NSm | | | COPD - Sm | | | Sm - NSm | | | Symbol |
|---|---|---|---|---|---|---|---|---|---|
| **FC** | **P.Value** | **adj.P.Val** | **FC** | **P.Value** | **adj.P.Val** | **FC** | **P.Value** | **adj.P.Val** | |
| 1.229549 | 0.00014 | **0.001944** | 1.144021 | 0.009588 | 0.097174 | 1.074761 | 0.044679 | 0.158584 | **TGFB1,** 203084_at |
| 1.26443 | 0.04929 | 0.137434 | 1.229588 | 0.072377 | 0.258710 | 1.028343 | 0.725102 | 0.847241 | **TGFB1, 203085_s_at** |
| 1.15446 | 0.00249 | **0.015944** | 1.117776 | 0.014651 | 0.117646 | 1.032823 | 0.30422 | 0.516196 | **TGFB2,** 220406_at |
| 1.06844 | 0.06018 | 0.158702 | 1.026389 | 0.441436 | 0.673560 | 1.040971 | 0.088401 | 0.244685 | **TGFB2, 209908_s_at** |
| 1.05489 | 0.07390 | 0.183367 | 1.061867 | 0.037767 | 0.185608 | -1.00661 | 0.741056 | 0.857235 | **TGFB2, 209909_s_at** |
| -1.0230 | 0.69866 | 0.817013 | 1.051098 | 0.380917 | 0.624023 | -1.07534 | 0.06681 | 0.205376 | **TGFB2,** 228121_at |
| -1.0112 | 0.75466 | 0.854673 | 1.046755 | 0.186139 | 0.42568 | -1.05851 | 0.018421 | 0.087290 | **TGFB2, 220407_s_at** |
| 1.04627 | 0.22355 | 0.393126 | 1.012978 | 0.718617 | 0.860221 | 1.032873 | 0.193904 | 0.395041 | **TGFB3, 1555540_at** |
| 1.01571 | 0.72436 | 0.833936 | 1.070529 | 0.111448 | 0.324411 | -1.05396 | 0.077238 | 0.224821 | **TGFB3, 209747_at** |
| 1.30718 | 0.00019 | **0.002401** | -1.0144 | 0.83345 | 0.922933 | 1.326 | 1.21E-08 | **7.60E-07** | **CTGF/CCN2, 209101_at** |
| -1.6201 | 2.01E-16 | **1.07E-13** | -1.10852 | 0.045117 | 0.203244 | -1.46153 | 8.21E-21 | **5.10E-18** | **LTBP1, 202729_s_at** |
| -1.0559 | 0.18056 | 0.341235 | 1.007871 | 0.841039 | 0.927261 | -1.06421 | 0.022821 | 0.101147 | **LTBP1, 202728_s_at** |
| 1.03525 | 0.59528 | 0.740602 | -1.08889 | 0.17699 | 0.414093 | 1.127284 | 0.006641 | **0.042327** | **BAMBI,** 203304_at |

**Bronchial Brushings GSE37147**

| COPD – NSm | | | COPD – Sm | | | Sm - NSm | | | Symbol |
|---|---|---|---|---|---|---|---|---|---|
| **FC** | **P.Value** | **adj.P.Val** | **FC** | **P.Value** | **adj.P.Val** | **FC** | **P.Value** | **adj.P.Val** | |
| -1.00059 | 0.98933 | 0.996489 | 1.048373 | 0.292529 | 0.657267 | -1.04899 | 0.16759 | 0.364401 | **TGFB1, 8037005** |
| 1.027413 | 0.68262 | 0.849468 | 1.001154 | 0.986292 | 0.995669 | 1.026229 | 0.617341 | 0.787117 | **TGFB2, 7909789** |
| -1.03943 | 0.15232 | 0.375008 | -1.01439 | 0.601946 | 0.854238 | -1.02469 | 0.24881 | 0.466996 | **TGFB3, 7980316** |
| 1.416326 | 7.40E-11 | **7.38E-09** | 1.16676 | 0.003203 | 0.076516 | 1.213896 | 2.24E-06 | **6.06E-05** | **CTGF/CCN2, 8129562** |
| -1.51442 | 1.08E-16 | **8.74E-14** | -1.06404 | 0.188704 | 0.547272 | -1.42328 | 5.50E-19 | **3.05E-16** | **LTBP1, 8041383** |
| 1.146229 | 0.00476 | **0.031258** | 1.077032 | 0.128292 | 0.460281 | 1.064248 | 0.098307 | 0.258028 | **BAMBI, 7926875** |

NSm – healthy non-smoker
Sm – healthy smoker
FC – Fold-change

*Online supplements are not copyedited prior to posting and the author(s) take full responsibility for the accuracy of all data.*

**e-Table 4. Previous studies on the expression of TGF-beta signaling pathway the bronchial wall and/or peripheral lung tissues of patients with stable COPD and control subjects.**

| Study | Subjects | treatments | Compartments | Principal Proteins investigated | Significant differences |
|---|---|---|---|---|---|
| Aubert et al. 1994 | 6 COPD (mild/mod) vs 6 C.S. | preoperative treatment: no glucocorticoids | Peripheral lung | TGFβ1 | any |
| Vignola et al. 1997 | 19 Chronic bronchitis (7 with COPD) vs 13 C.N.S. | untreated | Bronchial biopsies | TGFβ, GM- CSF,EGF | TGFβ↑ in E, SM; GM-CSF↑ in SM;EGF↑ in E |
| De Boer et al. 1998 | 14 COPD (mild/mod) vs 14 C.S. | untreated | Peripheral lung | TGFβ1, TGFRI,TGFRII | TGFβ1↑ in E and AE |
| Kokturk et al. 2003 | 13 COPD (mild/mod) vs 10 C.N.S. | untreated | Bronchial biopsies | TGFβ1 | TGFβ1→ in E, SM |
| Baraldo et al. 2005 | 12 COPD (mild/mod) vs 12 C.S. | untreated | Bronchial rings(glands) | TGFβ1, TGFβRII | TGFβ1→, TGFβRII↓ |
| Zandvoort et al. 2006 | 11 COPD (mild/mod) vs 8 COPD (sev./very severe) vs 8 C.S. | untreated | Peripheral lung | TGFβ1, TGFβRI, Smad2,3,4,7 | TGFβ1↓ E,St; TGFβRI↓E,St;Smad3,7↓ E,St |
| Zanini et al. 2009 | 10 COPD (mod./severe) vs 8 C.N.S. | Stable untreated patients | Bronchial biopsies | TGFβ | TGFβ↑all in SM |
| Soltani et al.2012 | 18 S-COPD (mild/mod) vs 13 ES-COPD (mils/mod) vs 15 C.S. vs 17 C.N.S. | Stable untreated patients | Bronchial biopsies | TGFβ1 in bm vessels | TGFβ1↑in CS, ES-COPD, S-COPD |

This table does not include the human studies performed in serum/plasma, sputum, bronchoalveolar lavage, and in "in-vitro" primary human bronchial and/or lung-isolated cells as well as animal models of COPD/emphysema.

Abbreviations: C.S., control smokers; C.N.S., control non-smokers; S-COPD, current smokers with chronic obstructive pulmonary disease (COPD); ES-COPD, ex-smokers with COPD; EGF, epidermal growth factor; GM-CSF, granulocyte-macrophage colony stimulating factor; AE, alveolar epithelium; E, epithelium; SM, submucosa; St, stroma; arrows (↑increase, ↓decrease, →similar value) indicate changes in diseased patients compared to control groups.

References: Aubert JD et al. Thorax 1994; 49:225-32; Vignola AM et al. Am J Respir Crit Care Med 1997;156:591-9; de Boer WI et al. Am J Respir Crit Care Med 1998;158:1951-7; Kokturk N et al. J Asthma 2003; 40:887-93; Baraldo S et al. Thorax 2005; 60:998-1002; Zandvoort A et al. Eur Respir J 2006; 28:533-41; Zanini A et al. Thorax 2009; 64:1019-24; Soltani A et al. PloS One 2012; /:e39736.

*Online supplements are not copyedited prior to posting and the author(s) take full responsibility for the accuracy of all data.*

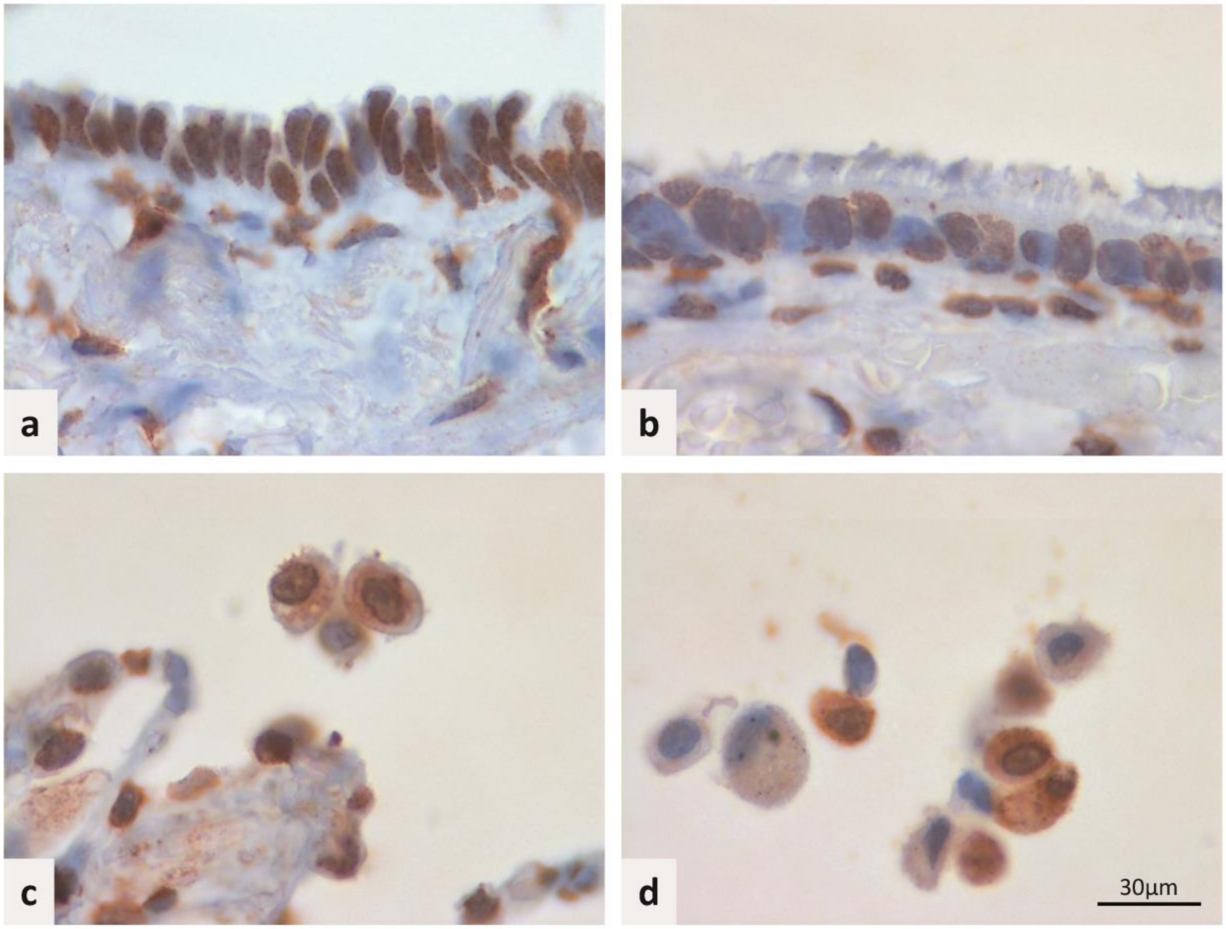


**e-Figure 1**
Photomicrographs showing the bronchiolar epithelium (panels a and b) and the alveolar macrophages (panels c and d) in smokers with normal lung function (panels a and c) and in stable COPD patients (panels b and d) immunostained for identification of TGFβ1. Results are representative of those from 12 control smokers and 12 COPD patients. Bar=30 microns.

*Online supplements are not copyedited prior to posting and the author(s) take full responsibility for the accuracy of all data.*

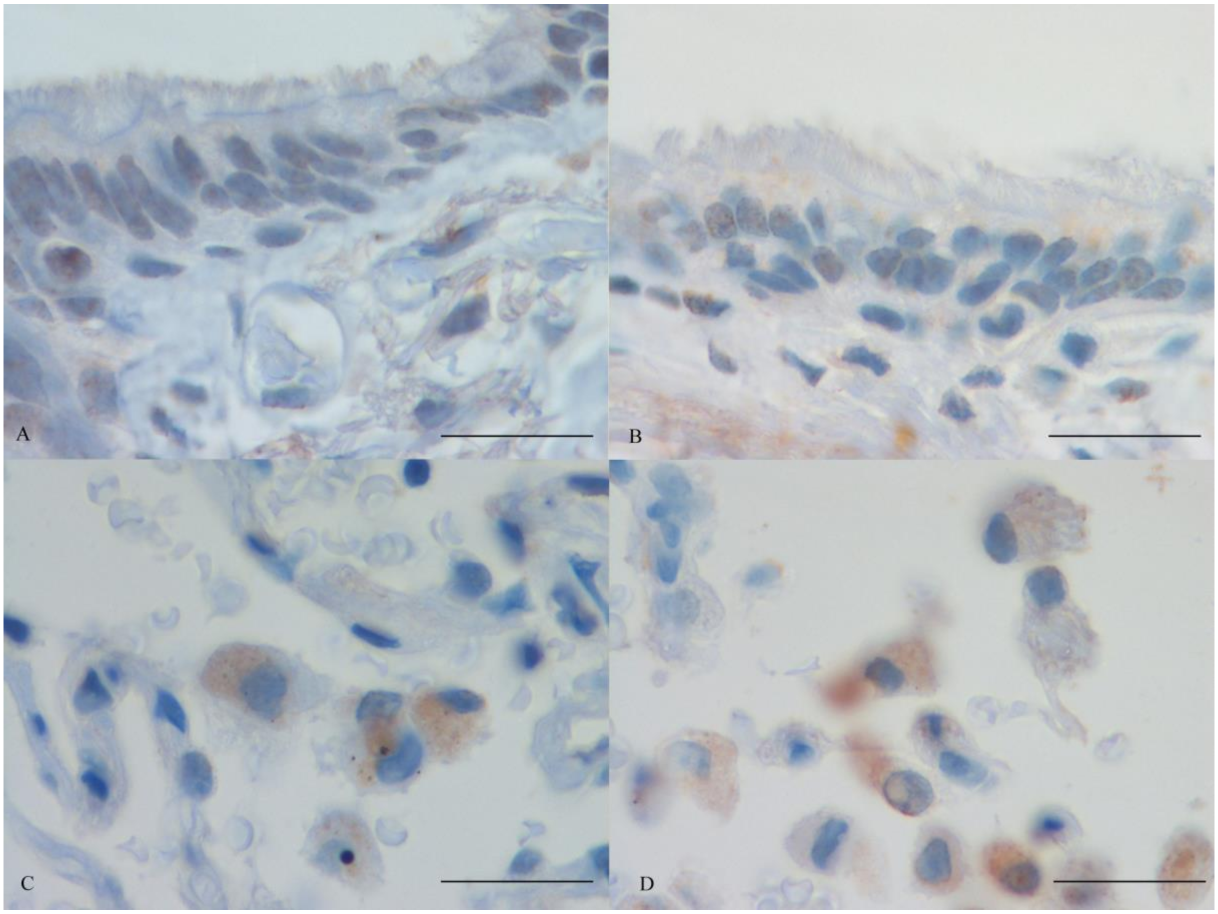


**e-Figure 2**
Photomicrographs showing the bronchiolar epithelium (panels a and b) in smokers with normal lung function (panel a) and in stable COPD patients (panel b) immunostained for identification of TGFβ3. Results are representative of those from 12 control smokers and 12 COPD patients. Bar=30 microns.

*Online supplements are not copyedited prior to posting and the author(s) take full responsibility for the accuracy of all data.*

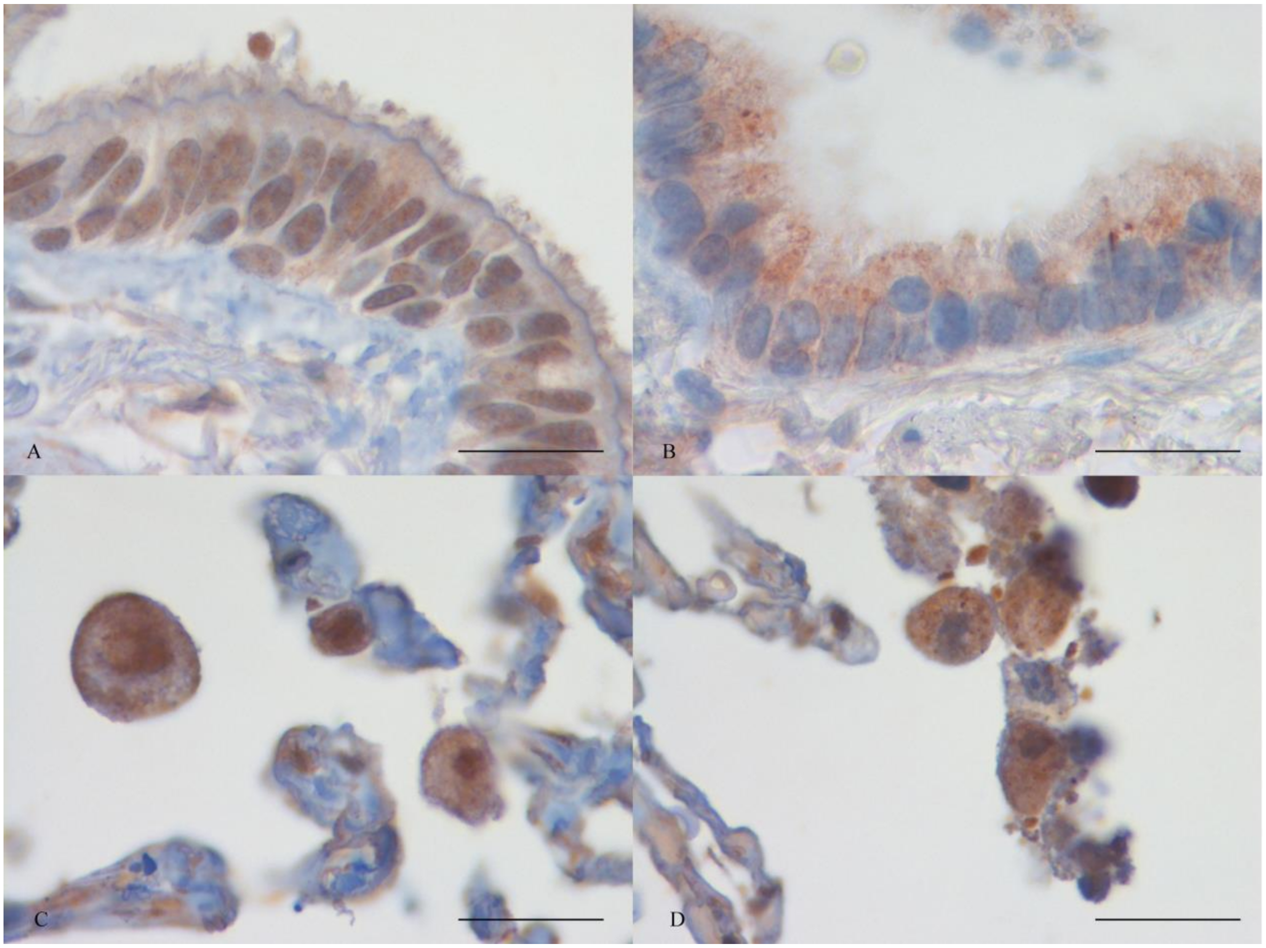


**e-Figure 3**
Photomicrographs showing the bronchiolar epithelium (panels a and b) in smokers with normal lung function (panel a) and in stable COPD patients (panel b) immunostained for identification of CCN2. Results are representative of those from 12 control smokers and 12 COPD patients. Bar=30 microns.

*Online supplements are not copyedited prior to posting and the author(s) take full responsibility for the accuracy of all data.*

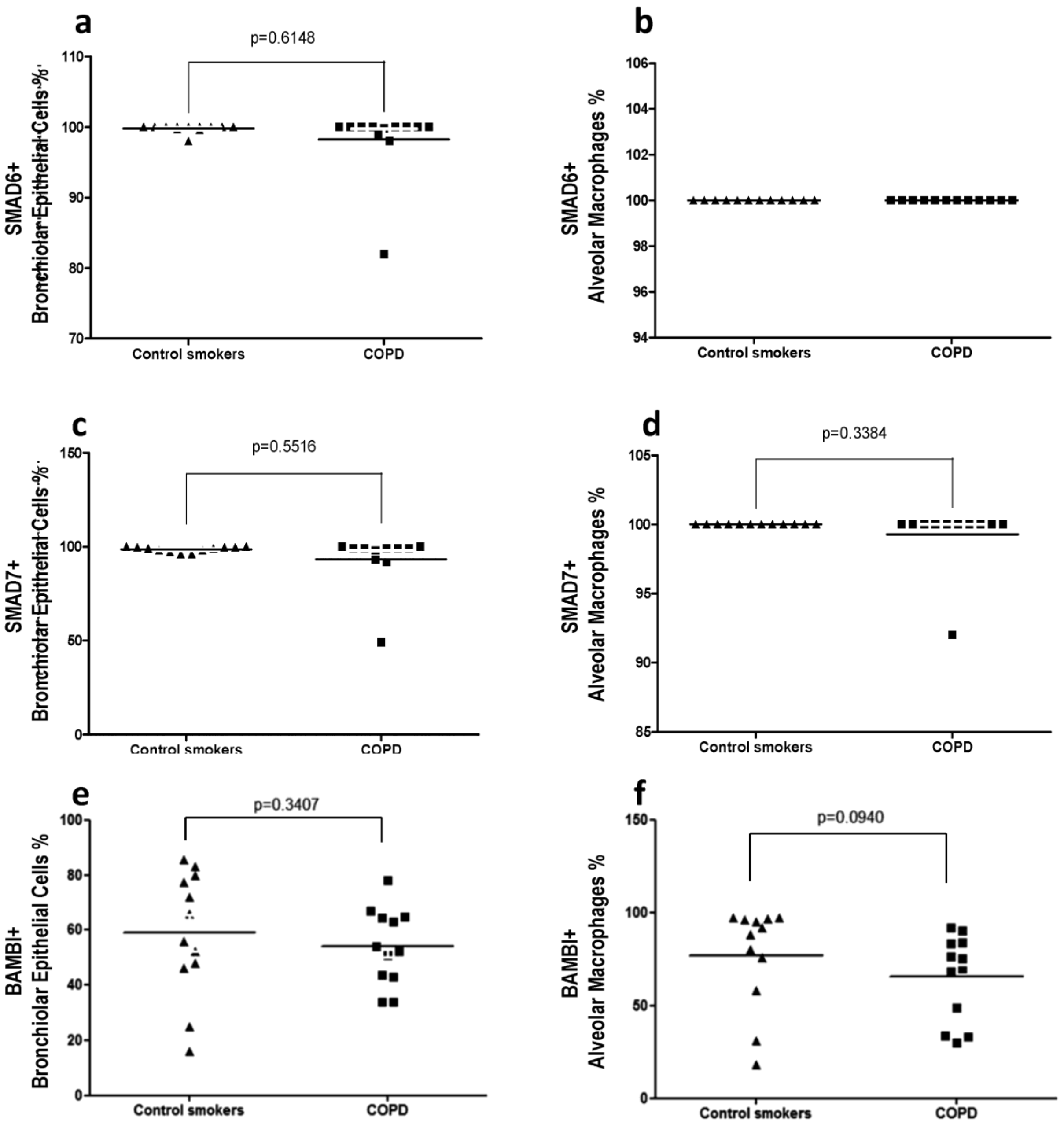


**e-Figure 4**

Graphical presentation of the percentage of the bronchiolar epithelial and alveolar macrophage cells immunostained for Smad6 (panels a and b, respectively), Smad7 (panels c and d, respectively) and BAMBI (panels e and f, respectively). No significant differences were observed between COPD patients and control smokers for any of the molecules studied in both compartments. Results are from 12 stable COPD and in 12 control smokers with normal lung function. Statistical analysis: Mann Whitney U test. Exact p values are shown above each graph.

Online supplements are not copyedited prior to posting and the author(s) take full responsibility for the accuracy of all data.

**Supplementary References**

S1. Vestbo J, Hurd SS, Agustí AG, et al. Global strategy for the diagnosis, management, and prevention of chronic obstructive pulmonary disease: GOLD executive summary. Am J Respir Crit Care Med. 2013;187:347-65.

S2. Global Initiative for Chronic Obstructive Lung Disease (GOLD): global strategy for the diagnosis, management and prevention of chronic obstructive pulmonary disease. NHLBI/WHO workshop report. NIH Publication No 2701A. goldcopd.org.

S3. Di Stefano A, Caramori G, Barczyk A, et al. Innate immunity but not NLRP3 inflammasome activation correlates with severity of stable COPD. Thorax. 2014;69:516-24.

S4. Marwick JA, Caramori G, Stevenson CS, et al. Inhibition of PI-3Kδ restores steroid responsiveness in smoking-induced steroid insensitivity. Am J Respir Crit Care Med 2009;179:542-8.

S5. Kirkham PA, Caramori G, Casolari P, et al. Oxidative stress-induced antibodies to carbonyl-modified protein correlate with severity of chronic obstructive pulmonary disease. Am J Respir Crit Care Med. 2011;184:796-802.



*Online supplements are not copyedited prior to posting and the author(s) take full responsibility for the accuracy of all data.*
